# MEG Connectivity and Power Detections with Minimum Norm Estimates Require Different Regularization Parameters

**DOI:** 10.1155/2016/3979547

**Published:** 2016-03-22

**Authors:** Ana-Sofía Hincapié, Jan Kujala, Jérémie Mattout, Sebastien Daligault, Claude Delpuech, Domingo Mery, Diego Cosmelli, Karim Jerbi

**Affiliations:** ^1^Psychology Department, University of Montreal, Montreal, QC, Canada H2V 2S9; ^2^Department of Computer Science, Pontificia Universidad Católica de Chile, 7820436 Santiago de Chile, Chile; ^3^School of Psychology and Interdisciplinary Center for Neurosciences, Pontificia Universidad Católica de Chile, 7820436 Santiago de Chile, Chile; ^4^Lyon Neuroscience Research Center, DyCog Team, Inserm U1028, CNRS UMR5292, 69675 Bron Cedex, France; ^5^Department of Neuroscience and Biomedical Engineering, Aalto University, 02150 Espoo, Finland; ^6^MEG Center, CERMEP, 69675 Bron Cedex, France

## Abstract

Minimum Norm Estimation (MNE) is an inverse solution method widely used to reconstruct the source time series that underlie magnetoencephalography (MEG) data. MNE addresses the ill-posed nature of MEG source estimation through regularization (e.g., Tikhonov regularization). Selecting the best regularization parameter is a critical step. Generally, once set, it is common practice to keep the same coefficient throughout a study. However, it is yet to be known whether the optimal lambda for spectral power analysis of MEG source data coincides with the optimal regularization for source-level oscillatory coupling analysis. We addressed this question via extensive Monte-Carlo simulations of MEG data, where we generated 21,600 configurations of pairs of coupled sources with varying sizes, signal-to-noise ratio (SNR), and coupling strengths. Then, we searched for the Tikhonov regularization coefficients (lambda) that maximize detection performance for (a) power and (b) coherence. For coherence, the optimal lambda was two orders of magnitude smaller than the best lambda for power. Moreover, we found that the spatial extent of the interacting sources and SNR, but not the extent of coupling, were the main parameters affecting the best choice for lambda. Our findings suggest using less regularization when measuring oscillatory coupling compared to power estimation.

## 1. Introduction

Healthy brain function is largely mediated by coordinated interactions between neural assemblies in different cortical and subcortical structures. As a result, the study of neuronal processes requires techniques that can reliably measure the spatiotemporal dynamics of large-scale networks. To this end, it is important to assess the modulations of local activations as well as long-range coupling between brain areas. Over recent years, the quantification of neuronal interactions in a given behavioral task or brain state has been the focus of a large body of research, and various techniques are currently used to detect and probe the role of functional connectivity (e.g., [1, 2]). In particular, a widely used technique for the detection of large-scale interactions among neural assemblies is magnetoencephalography (MEG) [3–5]. This is primarily due to its high temporal resolution, which is in the same order of magnitude as the neuronal processes themselves (milliseconds). Unlike electroencephalography (EEG), MEG measures magnetic fields that are less affected by the skull and brain tissue and provides whole-head coverage, which is mandatory for the assessment of large-scale brain networks. The use of MEG has advanced our comprehension of the mechanisms underlying functional brain connectivity [6] involved in sensory, motor, and higher-order cognitive tasks [3, 7–11] higher-order cognitive tasks resting state [6, 12–18].

The ability to measure brain interactions at the source level, rather than between sensor channels, is an important prerequisite if we want to make useful inferences about the anatomical-functional properties of the network. To this end, source reconstruction techniques are applied in order to estimate the spatiotemporal activity of the cortical generators underlying the recorded sensor-level MEG data. Solving this relationship is an ill-posed inverse problem for which numerous methods have been developed [4]. The differences between the various techniques mainly stem from the assumptions they make about the properties of the neural sources and from the way they incorporate various forms of a priori information, if any is available. Conceptually, solving the MEG inverse problem boils down to solving a system of equations that is underdetermined (i.e., no unique solution) since we generally have far more sources (thousands) than measurements (hundreds). Identifying a solution to such a problem can be achieved by imposing constraints on the sources. A typical constraint is to minimize the source power. Among these methods, the Minimum Norm Estimate (MNE) relies on minimizing the L2-norm and is one of the most widely used techniques [4, 7, 8, 18–37]. By contrast, estimates obtained by minimizing the L1-norm are referred to as Minimum-Current Estimates (MCE) [34, 38]. While the L2-norm assumes a Gaussian a priori current distribution, the L1-norm assumes a Laplacian distribution [39].

In principle, MNE looks for a distribution of sources with the minimum (L2-norm) current that can give the best account of the measured data. As the problem is ill-posed, MNE generally uses a regularization procedure that sets the balance between fitting the measured data (minimizing the residual) and minimizing the contributions of noise [22, 40, 41]. The Tikhonov or Wiener regularization [42, 43] and SVD truncation are among the most widely used regularization procedures. Regularization may be considered a necessary evil: it is required to stabilize the solution of the inverse problem, yet too much regularization leads to overly smooth solutions (spatial smearing). Since we do not have a precise model of brain activity and noise, the choice of the optimal amount of regularization is a nontrivial step for which no magical recipe exists [44]. The relationship between optimal regularization and the patterns of underlying generators is still poorly understood. In particular, the effect of regularization on the detection accuracy of Minimum Norm Estimates of source power and interareal source coupling has not been thoroughly investigated. This raises the question of whether one should use the same regularization coefficient for MNE-based power and connectivity analyses, as is generally done, or would one benefit from optimizing the regularization coefficients separately for each analysis? Furthermore, how does the optimal regularization coefficient, in such configurations, depend on sensor-level SNR, source size, or coupling strength?

With these questions in mind, we performed extensive Monte-Carlo simulations, creating over 20,000 pairs of coupled oscillatory time series in MEG source spaces, and computed the resulting surface MEG recordings. Then, we estimated source power and coherence using the MNE framework with variable degrees of Tikhonov regularization. Moreover, we used an approach based on an area under the curve (AUC) to identify the optimal regularization coefficient (lambda) in the case of power and coherence analyses. We found a systematic difference between the optimal lambdas in each analysis: for source-level coherence analysis the optimal lambda was two orders of magnitude smaller than the best lambda for power detection. Lastly, our findings are broken down as a function of SNR, cortical patch size, and corticocortical coupling strength.

## 2. Methods and Materials

In this section, we first present the MEG inverse problem formulation and the MNE framework. Then, we describe the simulation, reconstruction, and performance assessment procedures.

### 2.1. The MEG Inverse Problem

The inverse problem looks for an estimation of the active sources **S** (3*n*_sources_ × time  points) that generates the measurements **M** (*n*_channels_ × time  points) recorded at the sensors. The dimension 3*n* of the columns of **S** accounts for the three components of the source *n* in the *x*, *y*, and *z* directions. According to anatomical observations, the main generators of MEG are located in the grey matter and their orientation is perpendicular to the cortical sheet [45]. Here, we used a constrained orientation approach (perpendicular to the cortical surface), and hence the dimensions of **S** are reduced to *n*_sources_ × time  points. Assuming a linear relationship between the measurements and the active sources, the problem is modeled as

(1) $$\begin{array}{c} M=LS+N, \end{array}$$

where **L** is the lead field matrix (*n*_channels_ × *n*_sources_) and **N** is additive noise applied at the MEG channels (*n*_sources_ × time  points). The lead field matrix describes how each source contributes to the measurements at each sensor, given a specific head conductivity model and a source space. As the number of sources is usually much higher than the number of sensors, the lead field matrix is highly underdetermined and thus not invertible. The estimation of the activity of the sources requires the definition of an inverse operator **W**:

(2) $$\begin{array}{c} \hat{S}=W^{T}M, \end{array}$$

where $\hat{S}$ represents the estimated sources (*n*_sources_ × time  points) and the superscript *T* denotes matrix transpose.

### 2.2. Minimum Norm Estimate (MNE)

As the MEG inverse problem is ill-posed, a regularization scheme is needed [22], and one of the most common options is the Tikhonov regularization [42, 43]. MNE calculates an inverse operator **W** by searching for a distribution of sources $\hat{S}$ with minimum currents (L2-norm) that produces an estimation of the measurements ($L\hat{S}$) most consistent with the measured data (**M**). The solution is a trade-off between the norm of the estimated regularized sources current $\lambda^{2}{\left\|\hat{S}\right\|}^{2}$ and the norm of the quality of the fit they provide to the measurements ${\left\|M-L\hat{S}\right\|}^{2}$. Assuming the noise **N** and the sources strength **S** to be normally distributed with zero mean and covariance matrix **Q** and **R**, respectively, a general form of the MNE inverse solution can thus be given as [35, 46, 47]

(3) $$\begin{array}{c} \hat{S}=\underset{S}{\mathrm{argmin}}\left\{\;{\left\|\;Q^{-1/2}\left(\;M-LS\;\right)\;\right\|}^{2}+\lambda^{2}{\left\|\;R^{-1/2}S\;\right\|}^{2}\;\right\}, \end{array}$$

where *λ* is the Tikhonov regularization parameter. Thus, the inverse operator **W** is defined as

(4) $$\begin{array}{c} W=RL^{T}{\left(\;LRL^{T}+\lambda^{2}Q\;\right)}^{-1}, \end{array}$$

where the superscript −1 denotes matrix inverse. 

The Minimum Norm Estimate embodies the assumption of independently and identically distributed (IID) sources, which corresponds to an identity matrix **R** in the above formula. Alternatively, **R** can incorporate more informed (spatial) assumptions, yielding a so-called weighted Minimum Norm Estimate [28, 35, 44]. Here, we use the general and classical minimum norm solution. The noise covariance matrix **Q** was computed from the actual noise which was added to the sensors in each simulation.

### 2.3. Simulations

We simulated 117s of oscillatory activity in pairs of cortical sources with different degrees of coupling in the alpha band (9–14 Hz). We computed the resulting sensor-level data through forward modeling based on a 275-channel CTF MEG system configuration. Our sources consisted of current dipoles placed at the vertices of a tessellated MNI-Colin 27 cortical surface, which was segmented and tessellated using FreeSurfer [48] and downsampled to 15028 vertices. Different strengths of coupling were obtained by forcing the time series of the second source to have a certain level of coherence with the first source time series. The magnetic fields at the sensors were calculated using a single sphere head model and constraining the orientations of the sources to be normal to the cortical surface. The simulated data were generated using a combination of custom MATLAB code, in addition to functions from Brainstorm [49] and FieldTrip [50] toolboxes. The source time courses were simulated by first setting the base frequency of the oscillator (e.g., 12 Hz) and inducing a small jitter to its instantaneous frequency across time points. The frequency modulated time courses were then generated using an exponential function. This procedure causes fluctuations in the phase relationship between two oscillators with the same base frequency, allowing us to achieve coherence levels below 1. The frequency jittering was performed randomly in a loop until the desired coherence level (e.g., 0.4) between the time courses of the two oscillators was reached.

We randomly selected two locations (seeds) for each simulated pair of sources (600 pairs). Next, for each pair, we varied three additional parameters in the simulations: the spatial extent of the sources, the strength of the coupling between the two sources, and the signal-to-noise ratio (SNR) at the sensors. These parameters are described in more detail below.

*(i) Patches and Point-Like Sources*. We simulated point-like sources (i.e., 1 dipole) and cortical patches (with surface areas: 2, 4, or 8 cm^2^). The activity of a cortical patch was simulated by placing identical time series at the vertices that make up the patch. As described above, the vertices were obtained via tessellation of the MNI brain (Colin 27). 

*(ii) Coupling Strength*. When generating the time series for each of the two sources of a pair of simulated generators, we defined the coupling by setting the alpha-band coherence to either 0.1, 0.2, or 0.4. We simulated time series that were 7000 samples long (600 Hz sampling frequency). Note here that by actually simulating true coherence at the source level, we circumvent debates about spurious coupling that arises from field spread. As such, we assess here the ability to recover truly coherent cortical activity. 

*(iii) Signal*-*to*-*Noise Ratio (SNR)*. White noise was added to the sensor signals in order to achieve three levels of SNR (0 dB, −20 dB, and −40 dB), calculated as the ratio of the Frobenius norm of signal and noise amplitudes at the sensors.

In summary, we randomly chose 600 different pairs of cortical location configurations, for which we varied source size (4 levels: point-like, 2, 4, or 8 cm^2^), coupling strength (3 levels: 0.1, 0.2, or 0.4), and SNR (3 levels: 0 dB, −20 dB, and −40 dB). This yielded a total of 21,600 sets of simulated MEG sensor-level data. Next, we evaluated the effect of varying the Tikhonov regularization parameter *λ* on detection performance of MNE, independently, for power and for coherence mapping.

### 2.4. Power and Coherence Reconstructions

The previous section described the cortical power and coherence configurations that were generated in the MEG data simulation step. Now, in order to assess our ability to reconstruct these simulated (i.e., known) source configurations, we first reconstruct the source time series by applying MNE to the simulated sensor data, and then we compute spectral power and coherence from these estimated time series.

Most MEG studies that use MNE select a single lambda value once and for all to be used throughout the study; the same regularization coefficient is hence used for both power and coupling estimations (assuming both are performed within the study). This is not the case here, precisely because our objective is to test whether optimal lambda values differ between power and coherence mapping.

Therefore, in order to find the regularization coefficient providing the most accurate reconstruction of (a) power and (b) interareal coherence across all 21,600 simulated configurations, we used multiple values for *λ* ranging from 1*e* − 11 to 1*e* − 5 (7 values). In addition to searching for the optimal lambda that provides the best results across all simulations, the definition of best lambda was also separately examined as a function of SNR, coupling strength, and source extent. Finally, for the sake of comparison, we also computed the optimal *λ* value obtained from the standard L-curve method [51]. The range of possible lambda values examined in our estimations was selected based on visual inspection in a subset of simulations and chosen to ensure it included the lambda obtained via the L-curve approach.

Notably, for the coupling analysis, we used the reconstructed time series at one of the two simulated dipoles as reference point, and we calculated coherence with all other source time series across the brain. Spectral power (at any location *r*) and magnitude squared coherence (between two locations *r*_1_ and *r*_2_) were calculated as follows:

(5) Pr,f=Csr,r,f,Cohr1,r2,f=Csr1,r2,f2Pr1,r1,fPr2,r2,f,

where Cs is the cross-spectral density matrix and *f* is the frequency bin. Power and MS coherence were calculated via built-in standard MATLAB (Mathworks Inc., MA, USA) functions based on Welch's averaged and modified periodogram method [52].

### 2.5. Receiver Operating Characteristic (ROC) Curves

We evaluated the performance of each method using the area under the curve (AUC) from the ROC curves obtained by plotting the True Positive Fraction (TPF) versus the False Positive Fraction (FPF) at a given threshold *α*, calculated as follows:

(6) TPFα=TPαNumber  of  simulated  dipoles,FPFα=FPαTotal  number  of  dipoles−Number  of  simulated  dipoles,

where TP(*α*) represents the true positives (defined as the intersection between simulated sources and active sources at threshold *α*) and FP(*α*) represents the false positives (defined as all active sources but excluding the true positives at activation threshold *α*). By computing TPF and FPF repeatedly for successive values of activation thresholds *α*, we obtain a ROC curve from which we derive the AUC. The AUC is taken as a measure of performance; that is, the best regularization coefficient would be the one that yields the highest AUC. Statistical comparisons were performed using standard parametric two-tailed *t*-tests. Note that, for reference-based coherence reconstruction, computing true positives can be ambiguous if we consider the sources within the “reference patch” (location 1) to be true positives. To avoid this problem, we chose to quantify how well the distant coherent patch (location 2) was detected. In other words, we used the time series estimated at location 1 (as a seed) and considered only the simulated activity that make up the patch at location 2 to be the vertices we wish to detect. Therefore, the vertices within the reference patch were excluded from the ROC calculations for coherence evaluations.

## 3. Results

### 3.1. Optimal Tikhonov Regularization Coefficient Is Not the Same for Power and Coherence Reconstructions

**Figure 1:**
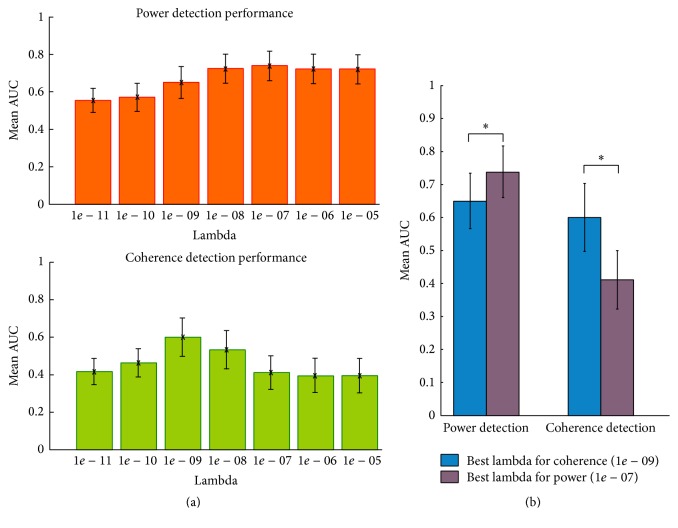
The averaged optimal regularization for power reconstruction is different from the one obtained for coherence detection. (a) Mean AUC for power and coherence reconstructions. (b) Mean AUC achieved with best lambda for power (1*e* − 07) or best lambda for coherence (1*e* − 09) when applied in each case both for power and for coherence. *∗* indicates statistically significant differences at *p* < 0.001, *t*-test.

Figure 1(a) shows the effect of lambda selection on quality of power and coherence detection across all simulated conditions and configurations (measured as mean AUC). Importantly, we found that the best mean value of lambda for power (10*e* − 7) differs from the best mean lambda for coherence, which was two orders of magnitude smaller (10*e* − 9). In comparison to the optimal value for power, the lower optimal value for coherence implies that a smaller residual should be allowed to have an optimal coherence reconstruction. The observations in Figure 1(a) also indicate that coherence reconstructions are more sensitive to the selection of an appropriate lambda value than power reconstructions (as AUC for coherence peaks at 1*e* − 09 and drops again, while AUC for power displays a flatter distribution for values neighboring 1*e* − 07).

Interestingly, Figure 1(b) shows a significant difference (*p* < 0.001, *t*-test) between the power reconstruction performances achieved using the optimal lambda for power compared to using the lambda determined to be optimal for coherence. Similarly, coherence reconstructions were also significantly better when using the coherence optimal ambda compared to the power optimal lambda. Simply put, our simulations demonstrate that, on average across 21,600 simulated pairs of sources, tuning lambda selection differently for coherence and for power analyses significantly impacts the results. In the next section, we examine how SNR, coupling strength, and source size individually affect these results.

### 3.2. Effect of SNR, Source Size, and Coupling Strength

**Figure 2:**
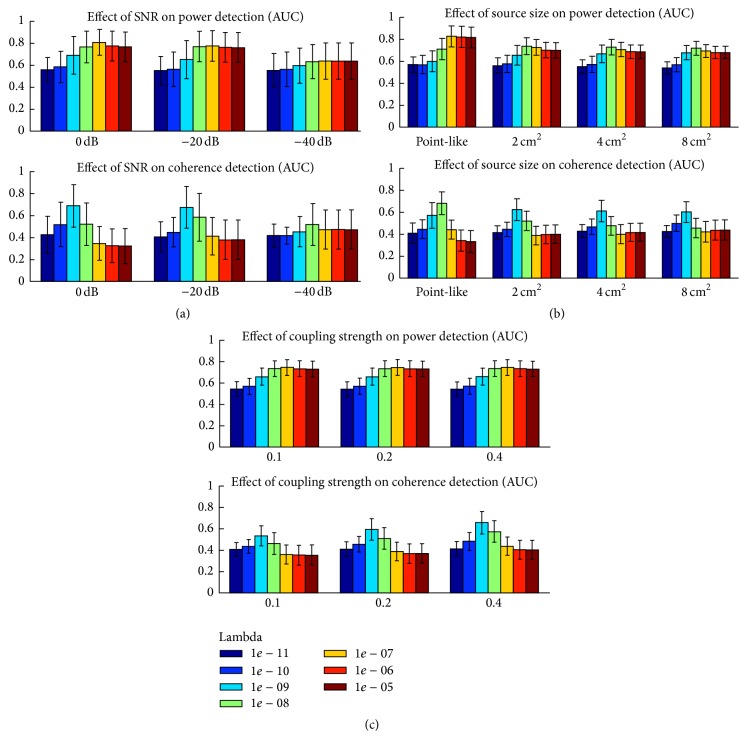
Power and coherence detection with MNE as a function of three simulation parameters: best lambda for power and coherence detection as a function of (a) SNR (0 dB, −20 dB, and −40 dB), (b) size of the sources (point-like, 2, 4, and 8 cm^2^), and (c) coupling strength (0.1, 0.2, and 0.4). Power and coherence performances are depicted in the upper and lower rows of each panel, respectively.

Figure 2 depicts the effect of (a) SNR, (b) source size, and (c) coupling strength on optimal lambda. In each panel, the results are shown independently for power (upper row) and coherence (lower row).

Figure 2(a) shows that a decrease in SNR leads, as expected, to an overall decrease in the performance of MNE as measured by mean AUC. For power reconstructions, a peak (i.e., easily identifiable optimal lambda) seems to be limited to the higher SNR. As for coherence reconstructions, the lower row of Figure 2(a) indicates that stronger regularization is needed as SNR drops. Next, Figure 2(b) suggests that for both power and coherence detection, point-like sources require more (an order of magnitude higher) regularization than cortical patches. Furthermore, Figure 2(c) shows that stronger coupling leads to better detection performances (higher mean AUC). However, it also suggests that the optimal lambda values (1*e* − 07 for power and 1*e* − 09 for coherence) do not vary with source coupling strength (Figure 2(c)).

### 3.3. Comparison with the L-Curve Method

A common and rather well-established heuristic to optimize *λ* in a data-driven manner is the L-curve. The L-curve is a plot of the norm of the regularization term versus the norm of the residuals, representing the trade-off between these two terms. The lambda with the best compromise between minimizing the norm of the current and that of the residual is chosen as the optimal lambda [51]. As it is a fast and widely used method, we decided to compare the optimal lambda given by the L-curve approach with the two average lambda values which we found to optimize either power or coherence.

**Figure 3:**
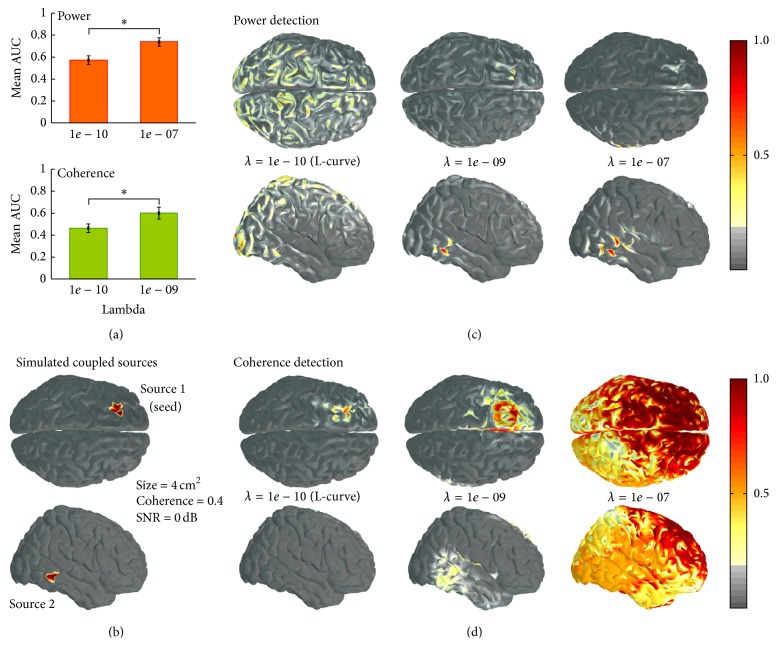
Differences in MNE performance when using the optimal lambda found using the L-curve approach and the best lambda values obtained from our simulations. (a) Difference in performance using the L-curve and the empirically derived lambda for power (upper row) and coherence (lower row) (*∗* indicates statistically significant differences at *p* < 0.001, *t*-test). (b) Illustrative example of a simulated pair of coupled sources (alpha-band coherence = 0.4, patch size = 4 cm^2^, and SNR = 0 dB). ((c) and (d)) Power and coherence reconstructions based on simulated data shown in panel (b) using three options for the definition of an optimal lambda value: *λ* = 1*e* − 10 (obtained with the L-curve), *λ* = 1*e* − 09 (mean optimal value for coherence detection), and *λ* = 1*e* − 07 (mean optimal value for power detection). Note that the power and coherence maps are normalized with respect to the maximum on each map and subsequently thresholded at 20% of maximum amplitude.

The mean optimal lambda obtained with the L-curve was 1*e* − 10. Application of this Tikhonov coefficient led to suboptimal reconstructions of both power and coherence. For both cases, the mean AUC obtained was significantly smaller (*p* < 0.001, *t*-test) than the mean AUC observed with the optimal lambda values derived from the data (Figure 3(a)). In fact, the L-curve based estimation of optimal lambda turned out to be one and three orders of magnitude smaller than the optimal values we had found for coherence and power, respectively. Figures 3(b)–3(d) show an example of a simulated pair of sources (Figure 3(a)) and its reconstructions using the optimal lambdas found with the data-driven AUC-based optimization (1*e* − 7 for power and 1*e* − 9 for coherence) and with the L-curve optimization (1*e* − 10). This configuration illustrates a typical case where the regularization coefficient derived from the L-curve approach fails. It also illustrates how the application of regularization that is suitable for power detection (1*e* − 07) can lead to very poor detection in the case of coherence, where less regularization (1*e* − 09) provides acceptable detection. Note that the case shown here is based on an arbitrary selection of a source configuration from among 21600 simulations. Both poorer and nicer single examples can be found of course, but we chose to represent a realistic intermediate case that serves to illustrate the point made by our study.

## 4. Discussion 

Overall, we have shown using extensive simulations of coupled pairs of sources that on average when using MNE, the best results are achieved by selecting separate regularization coefficients for power and for coupling estimations in source space. In particular, we found on average that the Tikhonov regularization coefficient that yields best coherence detection is substantially smaller than the optimal regularization coefficient for the detection of oscillatory power. This is largely due to the fact that increased smoothing (which arises from increased regularization) blows up the rate of false positives, a problem that appears to be amplified for corticocortical coupling.

Most MEG studies that apply MNE to estimate the source-level spatiotemporal dynamics do not fine-tune the regularization as a function of the proposed subsequent spectral analyses. Our simulations suggest that a regularization parameter that maximizes the detection of oscillatory source power may impair our ability to reliably reconstruct spectral connectivity. As a rule of thumb, we suggest using 1 to 2 orders of magnitude lower Tikhonov coefficient when searching for source coupling. Obviously, this suggestion stems from the simulations performed in this study and might not necessarily be the best choice if other minimum norm methods are used or if different source coupling configurations are present.

The variables that appear to have the strongest effect on the optimal lambda, according to our simulations, are the SNR and the spatial extent of the sources. In contrast, the strength of source coupling only minimally impacted the optimal choice for lambda. It is also noteworthy that, in general, hitting the right lambda seems to be even more critical for coherence than for power: while the performances for power appeared to stabilize when sufficient regularization is applied, coherence detection appeared to drop again when regularization becomes too excessive (see Figure 1(a)).

For comparison, we also used a standard procedure known as the L-curve approach to identify an optimal Tikhonov regularization from our simulated data. This resulted in a lambda value that was three orders of magnitude smaller than the best choice for power analyses and one order of magnitude smaller than the best choice for coherence analysis. The fact that the L-curve does not yield the best results is not a major problem. The L-curve approach remains a useful approach in real data analysis with MNE where the ground truth is not known. Here, because we simulated the MEG data, we were in a position to compare the performance of the regularization coefficient lambda calculated using the L-curve approach to the results achieved by a wide range of lambda values. Previous reports have also indicated suboptimal results when applying L-curve to simulated data (e.g., [53]). Other methods for a data-driven selection of lambda (e.g., [44, 47]) and other regularization methods (such as SVD truncation) have been proposed. For instance, lambda selection can be derived from SNR as lambda = trace(LRL^*T*^)/[trace(*Q*) × SNR^2^] [47] which with prewhitening and appropriate selection of source covariance matrix *R* (such that trace(LRL^*T*^)/trace(*Q*) = 1) yields lambda ~1/SNR, where SNR is (amplitude) signal-to-noise ratio. Approaches used to determine SNR values vary substantially. Exploring specific links between these formulations and the analyses performed here is of high interest, but it goes beyond the scope of the specific question we address, which focuses on differences in optimal lambda selection when switching between power and connectivity analysis.

Note that we focused here on MNE because it is a widely applied source estimation technique that is implemented in a number of toolboxes [49, 50, 54]. Besides, the classical minimum norm solution has been shown to be a valuable method whenever no reliable a priori information about source generators is available [30]. However, many other approaches exist (e.g., spatial filters) and the increased suitability of one method over another generally depends on the availability of a priori information and the validity, for the data at hand, of the theoretical assumptions that go into each method.

A prominent observation in the current study is that increases in the degree of regularization have a stronger impact on coherence than on power detection. While local power peaks remain fairly stable even with a high degree of smoothing, higher lambda values yield a drop in sensitivity in coherence analysis driven by an increase in false positive detections (spurious coupling). Since the smoothing effect is largely specific to MNE assumptions, one may ask whether coherence would be better estimated by using a different inverse approach based on different assumptions (e.g., spatial filters). While this could theoretically be the case, one should keep in mind that each inverse method comes with its own set of assumptions that are more or less suitable for the detection of coupled sources. For instance, from a theoretical point of view, the beamformer approach assumes that the sources are not correlated. In practice, beamformers are able to detect interacting sources if the extent of coupling is sufficiently weak. To fully tackle the question in the context of our data, we would need to perform the same simulation study using other inverse solutions alongside MNE in order to directly compare performances of the methods using the same detection metrics. This is part of an ongoing study and goes beyond the specific scope of the current paper which is to compare the effect of regularization on power and coherence detections using MNE.

It is noteworthy that our evaluation of coherence detection was characterized by how accurately the second source is detected when using the first as a seed point in a brain-wide coherence analysis. In other words, we do not use the result of a source localization procedure to identify the seed. Although this comes with certain limitations, it also allows us to address the two questions separately. In addition, corticocortical coupling is not exclusively performed on sources that show significant activations in the source localization step. A selection of a source or a region of interest (ROI) based on the literature (or on a specific hypothesis) is also used as a preliminary step to seed-based source-space coupling analyses. Our results directly apply to such approaches.

Although we explored many source configurations, varying spatial location, source coupling strength, SNR, and source size (21,600 simulations in total), our simulations are of course not exhaustive. For example, it could be of interest to extend this framework to EEG data, or a combination of EEG and MEG simulations (here we focused on MEG since it is more commonly used for source-level connectivity analysis). In addition, exploring more realistic noise signals and head models could be beneficial. Furthermore, it could be of interest to examine the effect of the presence of a third noninteracting source on our findings. Also, whether the results would significantly change if other forms of min-norm estimators are used is an open question, although previous findings suggest that this is quite unlikely [31].

Standard and modified ROC analyses and the associated AUC metrics have often been used to assess and compare the performance of different inverse methods to reconstruct EEG/MEG with simulated neural sources [47, 53, 55–58]. An alternative approach to comparing detection performance is the use of precision-recall (PR) curve [59]. This approach, as well as some of the modified ROC/AUC metrics mentioned above, can be particularly useful in the case of a heavily imbalanced ratio of true positives and true negatives (e.g., [60–62]). It has been shown that when comparing performances, a curve that dominates in ROC space will also dominate in PR space. However, a method that optimizes the area under the ROC curve is not guaranteed to optimize the area under the PR curve [63]. In the future, it could therefore be of interest to extend the current framework to include other performance metrics such as the PR curve.

Moreover, we restricted our coupling simulations to generating magnitude squared coherence between two distant time series. Coherence estimation has known limitations, such as its sensitivity to field spread effects in MEG [2]. Other coupling measures can be implemented in our framework in future studies, but it is important to keep in mind that we actually generated true magnitude squared coherence between the sources, and we evaluate the effect of regularization on our ability to recover this specific type of coupling from sensor recordings (in comparison to local spectral power). Likewise, an interesting question for future research is to evaluate to which extent these results hold in the presence of more than two coupled sources. In addition, all the results reported here pertain to the robustness of detecting absolute coherence and absolute power. It could be of interest to explicitly test the implications for condition-based (e.g., task A versus task B) or baseline-based (e.g., task A versus prestimulus period) comparisons. This being said, our results readily apply to analysis of ongoing MEG signals, such as MEG resting-state studies.

## 5. Conclusions

In this study, we address a simple question that has generally been overlooked in the field of MEG source reconstruction using MNE: should one use a different amount of regularization depending on whether one is interested in estimating local activity or in detecting interareal connectivity? Our results based on Monte-Carlo simulations (21,600 source configurations) suggest that optimal results are obtained when setting separate regularization coefficient, to estimate the source time series with MNE, for the analysis of power and coherence. In particular, coherence estimation in source-space is enhanced by using less regularization than what is used for spectral power analysis. Furthermore, we found that SNR and the spatial extent of the source generally have a stronger impact on lambda selection than coupling strength. These results provide some practical guidelines for MNE users and suggest, in particular, that one should regularize one to two orders of magnitude less when performing connectivity analysis, compared to local spectral power estimations. Ideally, if one plans to run both power and coupling analysis based on MNE source estimates, two distinct inverse operators should be implemented. Whether the phenomenon observed here may have similar implications or concerns in the context of other inverse solutions is an interesting topic for future research.
